# Impact of a direct-fed microbial supplementation on intestinal permeability and immune response in broiler chickens during a coccidia challenge

**DOI:** 10.3389/fmicb.2023.1283393

**Published:** 2023-10-31

**Authors:** Saheed Osho, Kevin Bolek, Kari Saddoris-Clemons, Brooke Humphrey, Miriam Garcia

**Affiliations:** Phibro Animal Health Corporation, Teaneck, NJ, United States

**Keywords:** broiler chickens, *Bacillus*, coccidia challenge, intestinal permeability, MicroLife Prime

## Abstract

Maintaining intestinal health supports optimal gut function and influences overall performance of broilers. Microlife^®^ Prime (MLP) contains a unique combination of four strains of *Bacillus* spp. selected to support a healthy gut which may improve performance. The aim of this study was to determine the effects of MLP supplementation on intestinal health and immunity of broilers challenged with a mixed coccidia infection during peak [0 to 6-day post-infection (dpi)] and recovery phases (6 to 13 dpi). A total of 120 male, 4 days-old Ross 708, broiler chicks were allotted to 3 treatment groups (8 replicate cages; 5 birds/cage) in a randomized complete block design. Treatments included a non-challenge (NEG), a coccidia challenge (POS), and coccidia challenge fed MLP (5 × 10^5^ CFU/g of diet). Diets were corn-soybean meal-based. At 11 days of age, all birds, except for NEG, were orally gavaged with 15 doses (3 × the recommended commercial dose). On 6, 9, and 13 dpi, birds were orally gavaged with fluorescein isothiocyanate conjugate dextran (FITC-d). Plasma and mid-jejunum tissues were collected 2 h later. On 6 dpi, duodenal lesions from 2 birds/cage were scored and droppings were collected for oocyst enumeration. Body weight gain (BWG) and feed conversion ratio (FCR) were calculated over the experimental period. Data were analyzed with GLIMMIX procedure of SAS. During the peak phase, POS birds had reduced BWG (23%) and FCR (15%) compared to NEG birds (*P* < 0.05), while birds fed MLP had similar BWG (209 and 208 g) and FCR (1.17 and 1.21) compared to NEG (*P* > 0.05). On 6 dpi, POS birds had higher lesion scores and oocyst shedding, 2 × increase in serum FITC-d, and higher jejunum IL-10, and IFN-_γ_ mRNA compared to NEG (*P* < 0.05). Birds fed MLP had reduced plasma FITC-d compared to POS birds (*P* < 0.05) and similar IL-10 and IFN-_γ_ mRNA. On 13 dpi, birds fed MLP had lower plasma FITC-d, jejunum IL-10 and IFN-_γ_ mRNA compared to POS birds (*P* < 0.05), but similar IL-10 to NEG birds (*P* > 0.05). This study confirms MLP improves intestinal health and positively modulates mucosal immune response post-coccidia challenge.

## 1. Introduction

Avian coccidiosis, a highly detrimental parasitic disease, poses significant challenges to the poultry industry, leading to millions of dollars in economic losses each year (Calik et al., 2019). This disease is characterized by *Eimeria* spp. colonization at multiple sites in the intestine leading to inflammation, tissue damage and diarrhea (Lee et al., 2010). Coccidiosis-related intestinal damage increases intestinal permeability and decreases growth performance (Dos Santos et al., 2020). Concerns about using ionophores anticoccidial/antibiotics in poultry production have grown due to resistance and residues in poultry products (Peek and Landman, 2003). As a result, antibiotic-free production has become more popular in the industry (Abdelrahman et al., 2014). Consequently, there is increased demand within the poultry industry for novel alternative strategies that improve performance and disease resistance. This includes methods to establish a robust intestinal barrier function, which is considered an essential factor in promoting the overall health and wellbeing of poultry (Jha et al., 2020).

In recent years, direct-fed microbials (DFM) have garnered significant attention for their beneficial effects on maintaining optimum gut health in poultry (Mackowiak, 2013; Grant et al., 2018). *Bacillus*-based DFMs have proven effective in broiler chickens, with a wide range of benefits, including growth promotion, intestinal epithelial cell proliferation, and gut barrier function (Ningsih et al., 2023). Over the past decade, the utilization of *Bacillus* species in broiler chickens has significantly increased, not only as growth enhancers but also as disease preventives (Sokale et al., 2019; Abd El-Hack et al., 2022; Yaqoob et al., 2022). Their high stability across extreme conditions ensures viability during manufacturing, processing, and storage, making *Bacillus* spores a promising and suitable feed additive to replace antibiotics (Ogbuewu et al., 2022).

Several studies in broiler chickens (Calik et al., 2019; Chasser et al., 2020; Nooreh et al., 2021) have primarily investigated efficacy of DFMs during the peak infective phase of *Eimeria* challenge, which occurs after a specific exposure period (day 4 to 7 post-infection). Peak infective phase is marked by a high prevalence of oocysts, extensive intestinal damage, and clinical signs of coccidiosis, including diarrhea, reduced feed intake, and compromised growth performance (El-Shall et al., 2022). However, limited research has been conducted on the recovery phase (day 9 to 14 post-infection) of *Eimeria* infection, which follows the peak infective stage and involves the host’s immune system controlling parasite growth and repairing damaged intestinal tissue. During this stage, oocyst shedding decreases, and clinical signs of coccidiosis reduce substantially (El-Shall et al., 2022). To address this research gap and broaden our understanding of the effects of DFMs on *Eimeria* infection, the objective of this study was to evaluate the effect of a commercial *Bacillus*-based DFM product on growth performance, intestinal permeability, lesion scores, oocyst shedding, and mRNA abundance of inflammatory cytokines during the peak and recovery phases following a mixed coccidia infection in broiler chickens. We hypothesized that dietary supplementation of a *Bacillus*-based DFM would ameliorate the impact of mixed coccidia infection during the peak and recovery phases in broiler chickens.

## 2. Materials and methods

### 2.1. Animal care

All animals were cared for under the guidelines outlined in the Phibro Animal Health Corporation, Animal Care and Use Policy. These guidelines follow the Guide for the Care and Use of Agricultural Animals in Research and Teaching (McGlone, 2010).

### 2.2. Product

A multi-strain *Bacillus*-based commercial direct-fed microbial (DFM) product (MicroLife^®^ Prime; Phibro Animal Health, Teaneck, NJ, USA) was used in this study. MicroLife Prime is a non-GMO direct-fed microbial product composed of four different *Bacillus* species (*B. subtilis*, *B. licheniformis*, *B. amyloliquefaciens*, and *B. coagulans*). *Bacillus* strains used in the MicroLife family of DFMs are naturally occurring and not subjected to genetic modification during the product manufacturing process.

### 2.3. Bird husbandry, experimental design, and treatments

A total of one hundred and twenty, 4-day old Ross 708, male broiler chicks were purchased from a commercial hatchery (Townline Hatchery, Zeeland, MI, USA). Upon arrival, all chicks were housed in two colony brooders (Universal Brooder Box, GQF Manufacturing, Savannah, GA, USA) and offered a broiler starter feed and water *ad libitum* for the first 2 days. The lighting schedule was 23L:1D throughout the experiment. Brooder and chick room temperatures were set to decrease as animals age, per commercial standards. All birds were provided with a corn-soybean meal basal diet in mashed form (free of antibiotics growth promoters and coccidiostats) formulated to meet or exceed the National Research Council (1994) nutrient recommendation for broiler chickens (Table 1). On day 4 post-hatch, 120 birds were individually tagged, weighed, and randomly assigned to 1 of 3 treatment groups in a randomized complete block design with 8 replicate cages per treatment and 5 birds per cage. Treatments included non-infected negative control (NEG) fed basal diet, a cocci challenge control fed basal diet (POS), and coccidia challenge fed basal diet supplemented with [DFM; MicroLife Prime (MLP, 5 × 10^5^ CFU/g of diet)]. Birds were fed experimental diets for 7 days prior to coccidia challenge.

**TABLE 1:** Ingredients and nutrient composition of basal diet, g/kg as-fed basis.

Ingredients	
Corn	523.0
Soybean meal (47% CP)	380.0
Soybean oil	50.0
Calcium carbonate (38% Ca)	15.0
Dicalcium phosphate	15.0
Salt	4.0
Vitamin-mineral premix^1^	5.4
_*DL*_-Methionine	3.8
_*L*_-Lysine.HCl	2.8
Threonine	1.0
Total	1,000
**Calculated nutrients and energy content**	
CP, g/kg	220
ME, kcal/kg	3,134
Ca, g/kg	9.2
P, g/kg	7.0
Ca:Tp	1.3

^1^Supplied the following per kilogram of diet: vitamin A, 5,484 IU; vitamin D_3_, 2,643 IU; vitamin E, 11 IU; menadione sodium bisulfite, 4.38 mg; riboflavin, 5.49 mg; _*D*_-pantothenic acid, 11 mg; niacin, 44.1 mg; choline chloride, 771 mg; vitamin B_12_, 13.2 μg; biotin, 55.2 μg; thiamine mononitrate, 2.2 mg; folic acid, 990 μg; pyridoxine hydrochloride, 3.3 mg; I, 1.11 mg; Mn, 66.06 mg; Cu, 4.44 mg; Fe, 44.1 mg; Zn, 44.1 mg; Se, 300 μg.

### 2.4. Coccidia infection, lesion scoring, and oocyst count

At 11 days of age [0-day post-infection (dpi)], birds from both the POS and DFM groups received an oral gavage of a live coccidia vaccine (Coccivac B52^®^, Merck, Madison, NJ, USA). The manufacturer recommends a dosage of 25 doses per kg BW. Given that a 1 kg bird would require 25 doses, a 200 g bird (the approximate weight at 11 days old) would typically receive 5 doses. However, for the purpose of challenging the birds, we administered 3 × the recommended amount, which equates to 15 doses per bird. Birds in the challenged groups (POS and DFM) were gavaged with 1 mL of coccidia vaccine containing the appropriate concentration (15 doses/bird) while birds in the NEG were gavaged with 1 mL of physiological saline. The vaccine contained live oocysts, isolated from chickens, and prepared from anticoccidia-sensitive strains of *E. acervulina*, *E. maxima*, *E. maxima MF*, *E. mivati*, and *E. tenella*. On 6 dpi, two birds per cage were randomly selected and euthanized for scoring of duodenal lesions caused by coccidia infection. Lesions in the duodenum were scored according to the method of Johnson and Reid (1970) by personnel blinded to treatment, based on scores ranging from 0 (no gross lesion) to 4 (most severe lesion). Droppings samples (∼100 g) were collected from each cage on 6 dpi and kept in separate airtight plastic bags. After homogenization, samples were stored at 4°C until assessed for oocyst counts, which were determined by dilution and counts via microscope using a McMaster counting chamber (Henriksen and Aagaard, 1976) and expressed as oocysts per gram of droppings.

### 2.5. Growth performance measurements

On 0, 6, and 13 dpi, body weight of birds and feed intake per cage were recorded to evaluate body weight gain and feed conversion ratio (FCR) over the experimental period.


FCR=weight⁢gain/feed⁢intake.

### 2.6. Gastrointestinal permeability

Fluorescein isothiocyanate dextran (FITC-d; MW 4kDa; Sigma-Aldrich Co., MO, USA) was administrated to evaluate intestinal leakage. The measurement of plasma FITC-d concentration was determined since FITC-d is a marker of mucosal barrier dysfunction. At four distinct time points-0 dpi (baseline, with 1 bird per cage), 6 dpi (2 birds per cage), 9 dpi (2 birds per cage), and 13 dpi (2 birds per cage)-birds were administered an oral dose of the FITC-d solution. “Each bird received specific amount of 500 μL of FITC-d solution,” and the specific concentration of the solution was calculated based on the bird’s body weight (BW) for each treatment group, which was 8.4 mg/kg BW. Two h following administration of the FITC-d on 0, 6, and 13 dpi, birds were euthanized by CO_2_ asphyxiation. Birds gavaged on 9 dpi were bled from wing vein and returned to their corresponding cages. Blood (6 mL) was collected into EDTA vacutainer tubes (BD company, Franklin Lakes, NJ, USA). Blood was kept on ice and then centrifuged at 2,000 × *g* at 4°C for 10 min to separate plasma. A standard solution was made by diluting FITC-d with a pool of plasma from 5 unchallenged birds at each time point. The FITC-d levels in the plasma samples and standard solution were measured at an excitation wavelength of 485 nm and an emission wavelength of 530 nm by using a microplate reader (BioTek Instruments, Inc., Winooski, VT, USA).

### 2.7. Jejunum collection and real-time polymerase chain reaction (PCR) analysis

A mid-jejunum sample was collected from 2 birds/cage on 6 and 13 dpi for quantitative real-time PCR (RT-qPCR) assay to quantify relative mRNA abundance of interferon-γ (IFN-_γ_), and interleukin-10 (IL-10). Samples were collected and flushed with PBS solution and ∼1 cm of the mid-jejunum was cut and immediately submerged in *RNAlater*^®^ stabilization solution (Thermo Fisher Scientific Inc., Waltham, MA, USA) and placed at room temperature for 1 h and then samples were stored at −80°C pending further analysis. Jejunal RNA was extracted using a Tissue Purification Kit (NORGEN BioTek Corp., Ontario, Canada), following manufacturer recommendations. Purity and concentrations of RNA samples were measured using a NanoDrop (Thermo Fisher Scientific). All samples had a 260/280 ratio between 1.83 and 2.11. Purified RNA was analyzed for the mRNA abundance of reference (GADPH, YWAHZ) and target (IFN-_γ_ and IL10) genes. Primers and TaqMan probes were obtained from Applied Biosystems (TaqMan^®^ Gene Expression Assays). Primers were run in duplex pairs (IL10-YWHAZ) or singlets (IFN-_γ_). Lack of primers cross-hybridization was evaluated by running reactions in singlet and pairs and confirming similar threshold cycle (Ct) values. For duplex reactions each primer was labeled with dyes with different fluorescence emission spectra (FAM or VIC). RT-qPCR reaction volumes consisted of 5 μL of RNA (40 ng/μL), 8 (duplex) or 9 (singlet) μL RNAse/DNase free water, 1 μL of each primer, and 5 μL Taqman Fast Virus 1-step Master Mix (cat no. 4444432 Applied Biosystems). All RT-qPCR reactions were conducted in a CFX96 optics unit mounted on a C1000 touch base (BioRad, Hercules, CA, USA) using the Fast-cycling mode recommended by the manufacturer. Samples were run in duplicate and rerun if the SD between Ct duplicates was > 0.3. The Ct-values from target genes were normalized to the geometric mean of the two reference genes Ct-values. Relative mRNA abundance was calculated using the 2^–ΔΔ^ ^CT^ method (Livak and Schmittgen, 2001). Results were expressed as fold-change relative to NEG treatment groups.

### 2.8. Statistical analysis

Data were analyzed using the GLIMMIX procedure of SAS (SAS Inst., Inc., Cary, NC, USA) with the fixed effect of treatment and the random effect of block (Initial BW). Experimental unit was the cage. The following statistical model was used in the analysis: Y_ij_ = μ + W_i_ + B_j_ + ε_ij_ where Y is the response criterion; μ is the overall mean; Wi is the effect of ith treatment (*i* = 1, 2, and 3); B_j_ is the effect of jth block (*k* = 1, 2, 3,…8); and ε_ij_ is the error term. All data were tested for normal distribution using Shapiro–Wilk test in PROC UNIVARIATE; oocyst count data were natural log transformed to achieve normality. Significant difference was defined as *P* ≤ 0.05.

## 3. Results

### 3.1. Growth performance

Prior to coccidia challenge, broiler chicks were fed the dietary treatments for 7 days. Growth performance did not differ between treatments from day 0 to 7 (data not shown). The effect of treatments on growth performance during a coccidia challenge is summarized in Table 2. During the peak phase (0–6 dpi), body weight (BW) and BW gain of birds in the POS group were significantly reduced (*P* < 0.05) as compared to birds in NEG group, whereas birds fed DFM had similar BW, BW gain and feed conversion ratio (FCR) when compared to birds in NEG group. However, there were no differences in feed intake across all treatment groups. Furthermore, during the recovery phase (7–13 dpi) there were no differences in BW, BW gain and FCR between treatment groups. However, we observed a higher (*P* < 0.05) feed intake for birds in NEG when compared to POS and DFM group.

**TABLE 2:** Growth performance, oocyst shedding, and lesion scores in broiler chickens fed diets supplemented with or without *Bacillus*-based direct-fed microbials (DFM) during a coccidia challenge.^1^^,^^2^

	Uninfected	Coccidia infection			
**Item**	**NEG**	**POS**	**DFM**	**SEM**	***P*-value**
**0–6 dpi (peak phase)**					
Final body weight, g	409^a^	358^b^	407^a^	10.1	< 0.01
BW gain, g/bird	209^a^	160^b^	208^a^	6.06	< 0.01
Feed intake, g/bird	246	220	252	20.9	0.41
Feed conversion ratio	1.17^b^	1.37^a^	1.21^b^	0.04	0.03
**7–13 dpi (recovery phase)**					
Final body weight, g	833	756	820	18.7	0.07
BW gain, g/bird	424	398	414	12.2	0.38
Feed intake, g/bird	464^a^	402^b^	384^b^	9.8	0.04
Feed conversion ratio	1.09	1.01	0.92	0.03	0.65
**Oocyst per gram of droppings (× 10^2^)**					
6 dpi	0^c^	47^a^	25^b^	4.0	0.03

^a–*c*^Means in the same row without common superscript differ significantly (*p* < 0.05).

^1^Data are least squares means of 8 replicate cages per treatment.

^2^Non-infected negative control (NEG) fed basal diet, a coccidia challenge positive control (POS) fed basal diet, and DFM [cocci challenge fed basal diet supplemented with MicroLife Prime (5 × 10^5^ CFUs/g of diet)]. On day 11 post-hatch [0-day post-infection (dpi)], birds in the POS and DFM groups were orally gavaged with 3 × the recommended coccidia vaccine (Coccivac B52^®^, Merck, Rahway, NJ, USA). Birds in the challenged group were gavaged with 1 mL of coccidia vaccine containing the appropriate concentration while birds in the NEG were gavaged with 1 mL of physiological saline.

### 3.2. Gastrointestinal permeability

**FIGURE 1:**
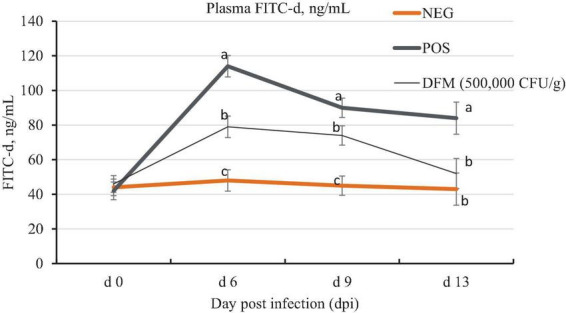
Plasma fluorescein isothiocyanate dextran (FITC-d) concentration of broiler chickens fed diets supplemented with or without *Bacillus*-based direct-fed microbial (DFM) during a coccidia challenge. Data are least squares means of 8 replicate cages per treatment. ^a–*c*^Means in the same dpi without common superscript differ significantly (*p* < 0.05). Non-infected negative control (NEG) fed basal diet, a coccidia challenge positive control (POS) fed basal diet, and DFM [cocci challenge fed basal diet supplemented with MicroLife Prime (5 × 10^5^ CFUs/g of diet)]. On day 11 post-hatch [0-day post-infection (dpi)], birds in the POS and DFM groups were orally gavaged with 3 × the recommended coccidia vaccine (Coccivac B52^®^, Merck, Rahway, NJ, USA). Birds in the challenged group were gavaged with 1 mL of coccidia vaccine containing the appropriate concentration while birds in the NEG were gavaged with 1 mL of physiological saline.

The dynamic change of gastrointestinal permeability from 0, 6, 9, and 13 dpi is shown in Figure 1. The results of gastrointestinal permeability are represented as level of FITC-d recovered in the plasma of birds. Higher concentration of FITC-d in the plasma represents an increase of gastrointestinal permeability. On 0 dpi, there were no significant (*P* > 0.05) differences among treatments. Birds challenged with coccidiosis (POS and DFM) had significantly higher (*P* < 0.05) plasma FITC-d concentration compared to the NEG birds on 6 and 9 dpi. Birds fed the DFM, however, had significantly lower (*P* < 0.05) plasma FITC-d concentration on 6, 9, and 13 dpi compared to the NEG birds. However, on 13 dpi there was still a significantly higher (*P* < 0.05) FITC-d in POS group when compared with the NEG group. The most severe gastrointestinal permeability was observed on 6 dpi across all treatment groups.

### 3.3. Lesion scores and oocyst count

**FIGURE 2:**
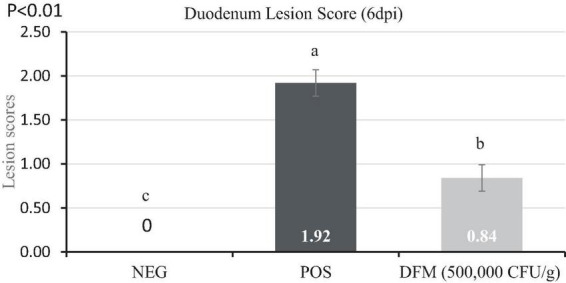
Lesion scores of broiler chickens fed diets supplemented with or without *Bacillus*-based direct-fed microbial (DFM) during a coccidia challenge. Data are least squares means of 8 replicate cages per treatment. ^a–*c*^Means in the same dpi without common superscript differ significantly (*p* < 0.05). Non-infected negative control (NEG) fed basal diet, a coccidia challenge positive control (POS) fed basal diet, and DFM [cocci challenge fed basal diet supplemented with MicroLife Prime (5 × 10^5^ CFUs/g of diet)]. On day 11 post-hatch [0-day post-infection (dpi)], birds in the POS and DFM groups were orally gavaged with 3 × the recommended coccidia vaccine (Coccivac B52^®^, Merck, Rahway, NJ, USA). Birds in the challenged group were gavaged with 1 mL of coccidia vaccine containing the appropriate concentration while birds in the NEG were gavaged with 1 mL of physiological saline.

Lesion scores and oocyst count are presented in Figure 2 and Table 2, respectively. Dietary DFM supplementation significantly reduced (*P* < 0.05) lesion scores and oocyst shedding in the duodenum when compared with the POS group. No lesions and oocyst shedding were observed in the NEG birds indicating birds remained free of coccidiosis infection.

### 3.4. Jejunal mRNA expression analysis

Cytokine mRNA expression in the mid-jejunum of broiler chickens was analyzed to assess differences in host immune responses between treatment groups (Table 3). At the peak phase (6 dpi), birds challenged with coccidia vaccine (POS and DFM) had significant upregulation (*P* < 0.05) of IL-10 and IFN-_γ_ when compared to the NEG group. During the recovery phase, IL-10 and IFN-_γ_ remained up-regulated (*P* < 0.05) in the POS group when compared to birds in NEG group. However, birds fed DFM had similar IL-10 mRNA expression as the NEG group and an intermediate up-regulation of IFN-_γ_, with expression lower than POS group but higher than NEG group (*p* < 0.05).

**TABLE 3:** Relative mRNA expression of cytokines in mid-jejunum tissue of broiler chickens fed diets with or without *Bacillus*-based direct-fed microbial (DFM) during a coccidia challenge (dpi).^1^^,^^2^

	Uninfected	Coccidia infection			
**Item**	**NEG**	**POS**	**DFM**	**SEM**	***P*-value**
**6 dpi (peak phase)**					
IL-10	1.00^b^	2.39^a^	2.14^a^	0.22	0.04
IFN-_γ_	1.00^b^	3.13^a^	2.91^a^	0.24	< 0.01
**13 dpi (recovery phase)**					
IL-10	1.00^b^	2.03^a^	1.45^b^	0.06	0.02
IFN-_γ_	1.00^c^	2.73^a^	1.91^b^	0.35	0.04

^a–*c*^Means in the same row without common superscript differ significantly (*p* < 0.05).

^1^Data are least squares means of 8 replicate cages per treatment. IL-17, interleukin-17; IL-10, interleukin-10; interferon-gamma, IFN-_γ_.

^2^Non-infected negative control (NEG) fed basal diet, a coccidia challenge positive control (POS) fed basal diet, and DFM [cocci challenge fed basal diet supplemented with MicroLife Prime (5 × 10^5^ CFUs/g of diet)]. On day 11 post-hatch [0-day post-infection (dpi)], birds in the POS and DFM groups were orally gavaged with 3 × the recommended coccidia vaccine (Coccivac B52^®^, Merck, Rahway, NJ, USA). Birds in the challenged group were gavaged with 1 mL of coccidia vaccine containing the appropriate concentration while birds in the NEG were gavaged with 1 mL of physiological saline.

## 4. Discussion

The search for efficient non-antibiotic alternatives to mitigate intestinal pathogens in poultry production continues to be a work in progress. Probiotics, or direct-fed microbials (DFM), have been utilized as non-antibiotic feed alternatives to promote beneficial bacteria establishment and modulate intestinal microbiome, ultimately enhancing gut barrier function and immune response. The current study delved into exploring the impacts of dietary *Bacillus*-based DFM supplementation on intestinal integrity and immune response in broiler chickens subjected to a direct coccidia challenge. During all phases of the study, the coccidia infection had a negative effect on growth performance. On average, the body weight (BW) gain was reduced by 15% and feed conversion was increased by 20% in the POS group from 0 to 13 dpi. This outcome was expected as coccidia infections are known to cause significant damage to the intestinal mucosa and enterocytes, leading to impaired nutrient absorption and a decline in performance (Dunaway and Adedokun, 2021; Herrero-Encinas et al., 2021). Additionally, parasitic infections trigger a reallocation of nutrient resources from growth to immune response, further contributing to growth variations (Amerah and Ravindran, 2015; Rochell et al., 2016). The present study demonstrated that dietary DFM supplementation alleviated the negative effect of coccidia infection on BW gain during both the peak and recovery phases. Several studies (Knap et al., 2011; Tan et al., 2014; Abudabos et al., 2018) have provided abundant evidence demonstrating the growth-promoting benefits of probiotics, irrespective of whether the conditions were pathogen-infected or non-infected. This can be attributed to various compounds secreted by *Bacillus*-based DFM, including digestive enzymes, antibacterial substances, and other growth-promoting factors such as short-chain fatty acids (Lee et al., 2015; Nusairat et al., 2018).

Birds in the POS group exhibited damage to the gut epithelium as evidenced by high lesion scores, while birds fed diets including the DFM showed lower lesion scores at 6 dpi. Our findings support those of Lee et al. (2010) who found that birds administered a *Bacillus*-based DFM strain exhibited significantly lower lesion scores in the gastrointestinal tract compared to birds fed a non-supplemented diet, particularly following an *E. maxima* challenge. The presence of fewer lesion scores indicates reduced damage to the intestinal epithelium, thereby increasing the likelihood of infected birds recovering from the disease. This reduction in intestinal lesion scores may be attributed to *Bacillus* probiotics’ ability to eliminate enteric pathogens through competitive exclusion and antagonisms (Calik et al., 2019).

Fecal oocyst count has been widely used as an indirect indicator of the severity of coccidia infection in broiler chickens (Leung et al., 2019; Chasser et al., 2020). In this study, as well as in previous studies (Lillehoj et al., 2016; Park et al., 2021), birds fed the DFM had a significantly lower oocyst shedding compared to birds in the POS group. This observed reduction in oocyst shedding provides compelling evidence of DFM’s multifaceted abilities in combating coccidia infection.

Throughout the current experiment, we closely monitored the dynamic changes in gastrointestinal permeability from 0 to 13 dpi to gain a comprehensive understanding of intestinal leakage during coccidia infection. Prior to coccidia infection (0 dpi), the intestinal permeability or integrity of the epithelial layer was intact across all treatment groups. However, at 6, 9, and 13 dpi, the birds in the POS group exhibited significantly higher FITC-d concentration compared to the NEG and DFM groups. This finding aligns with a previous report by Teng et al. (2020), which also observed increased intestinal permeability at 5, 6, 7, and 9 dpi following an *Eimeria* challenge. Interestingly, the DFM group demonstrated a noteworthy decrease in plasma FITC-d concentration compared to the POS group at all timepoints, indicating an improvement in intestinal integrity. This suggests that the DFM treatment contributed to preserving the gut barrier function and mitigating the increase in intestinal permeability typically associated with *Eimeria* infection. Our findings using FITC-d to assess intestinal permeability suggest that by 13 dpi, birds in the DFM group during coccidia challenge achieved intestinal integrity similar to that of birds in the NEG group. This indicates a recovery of intestinal integrity for the DFM-fed birds at 13 dpi, in contrast to the POS group, which had not fully recovered by the same time point. This highlights the potential role of reduced intestinal permeability in contributing to the improved gut health and recovery observed in DFM-fed birds.

To delve deeper into the protective effect of *Bacillus*-based DFM supplementation against coccidia challenge, our study also focused on assessing the mRNA abundance of cytokines in mid-jejunum tissue at the peak and recovery phases. As highlighted in previous research, *Eimeria* parasites invade epithelial cells triggering local inflammatory responses in broiler chickens (Kim et al., 2019). Cytokines, being immune regulatory peptides facilitating cell communication during immune responses, serve as recognized biomarkers for intestinal health (Park et al., 2021). As reported in our result, no differences were observed between POS and DFM groups in terms of IFN-γ and IL-10 expression levels at the peak phase, however, at the recovery phase, there was a decreased expression of IFN-_γ_ and IL-10 in DFM group when compared to POS group. As early as 3 dpi, elevated levels of IFN-_γ_ has been observed in response to *Eimeria* infections in chickens (Schneider et al., 2001; Hong et al., 2006). Recent evidence indicates that DFM’s may enhance host defenses against infection because of the bacteria’s effect on host immunity and gut integrity under enteric pathogen challenge (de Oliveira et al., 2019; Šikić Pogačar et al., 2020). Previous work by Rajput et al. (2013) reported that a *Bacillus*-based DFM significantly induced inflammatory and anti-inflammatory cytokines in jejunum and ileum of broiler chickens. Our research findings indicated the DFM group showed a quicker recovery to baseline after the challenge, likely due to a milder infection, as indicated by reduced lesions and oocyst shedding. Consequently, the immune system required less time to clear the infection. Notably, the DFM supplementation protected the gut mucosa, leading to a reduced workload for the immune system compared to scenarios involving active immune modulation. Our results establish a significant association between cytokine expression and gastrointestinal permeability during the recovery period. The damage caused on the intestinal mucosa by coccidia infection triggers robust inflammatory responses, leading to alterations in intestinal permeability and development of “leaky gut” conditions (Gilani et al., 2016). The decreased mRNA abundance of IL-10 and IFN-_γ_ observed at 13 dpi may be attributed to the mitigated intestinal leakage achieved through DFM supplementation at similar time point. This conclusion finds further support in the decreased IFN-_γ_ at the recovery phase in DFM-fed birds compared to birds in NEG group, illustrating their crucial role in regulating immune response against coccidia infection at recovery phase. Moreover, the significantly decreased expression of the anti-inflammatory cytokine IL-10 (primarily Th2-related) expression in DFM-fed group at the recovery phase compared to POS group, reinforces the notion that a *Bacillus*-based DFM induces a rapid recovery after a mixed coccidia infection. As produced by activated macrophages, IL-10 plays a key immunoregulatory role, primarily involved in controlling innate immune reactions (Rochell et al., 2017). This fine balance in controlling host innate immune responses upon coccidia infection is essential to prevent severe inflammation that may result in collateral damage to the intestine. Overall, the immunomodulatory effects of DFM-fed birds during the recovery phase of a mixed coccidia infection emphasize its potential as a valuable strategy to promote intestinal health and enhance resilience against pathogenic challenges in broiler chickens.

In conclusion, our study demonstrates that DFM supplementation effectively restored intestinal permeability following a coccidia challenge and provided overall protective effects during a coccidia infection. Specifically, our findings support its capacity to (1) effectively impact the small intestinal without compromising its functional activity, (2) to reduce parasite fecundity, and (3) alleviate the severity of gut damage associated with coccidia infection. Therefore, the decreased presence of intracellular pathogens in DFM-supplemented birds attests to the establishment of a healthier intestine with minimal epithelial damage. The observed improvements in growth performance, intestinal health, and immune response underscore the potential benefits of MicroLife Prime as a promising non-antibiotic alternative to mitigate coccidia infection in poultry production.

## Data availability statement

The original contributions presented in this study are included in this article/supplementary material, further inquiries can be directed to the corresponding author.

## Ethics statement

The animal study was approved by the Phibro Animal Health Corporation, Animal Care and Use Policy. The study was conducted in accordance with the local legislation and institutional requirements.

## Author contributions

SO: Writing – original draft, Writing – review and editing. KB: Writing – review and editing. KS-C: Writing – review and editing. BH: Writing – review and editing. MG: Writing – review and editing.
